# Arousal Predisposition as a Vulnerability Indicator for Psychosis: A General Population Online Stress Induction Study

**DOI:** 10.1155/2015/725136

**Published:** 2015-06-23

**Authors:** Annika Clamor, A. Malika Warmuth, Tania M. Lincoln

**Affiliations:** Department of Clinical Psychology and Psychotherapy, University of Hamburg, Von-Melle-Park 5, 20146 Hamburg, Germany

## Abstract

Explanatory models ascribe to arousability a central role for the development of psychotic symptoms. Thus, a disposition to hyperarousal (i.e., increased arousal predisposition (AP)) may serve as an underlying vulnerability indicator for psychosis by interacting with stressors to cause symptoms. In this case, AP, stress-response, and psychotic symptoms should be linked before the development of a diagnosable psychotic disorder. We conducted a cross-sectional online study in a population sample (*N* = 104; *M*_age_ = 27.7 years, *SD* = 11.2, range 18–70). Participants rated their AP and subclinical psychotic symptoms. Participants reported their stress-levels before and after two stress inductions including an arithmetic and a social stressor. The participants with an increased AP generally felt more stressed. However, AP was not associated with the specific stress-response. As expected, positive psychotic symptoms were significantly associated with AP, but this was not mediated by general stress-levels. Its association to subtle, nonclinical psychotic symptoms supports our assumption that AP could be a vulnerability indicator for psychosis. The trait is easily accessible via a short self-report and could facilitate the identification of people at risk and be a promising target for early stress-management. Further research is needed to clarify its predictive value for stress-responses.

## 1. Introduction

Psychotic symptoms such as blunted affect, hallucinations, and delusions may sometimes appear as being very distinct from usual perception and behavior. However, research indicates that “normal” personality traits such as neuroticism precede these symptoms and moderate emotional experience in psychosis [1]. As personality traits influence the perception of and coping with environmental challenges, it seems intuitive to assume that particular traits may constitute vulnerability indicators for the development of psychotic symptoms by interacting with those challenges. The mechanism as such has been postulated by vulnerability-stress-models of psychosis [2, 3], yet research on distinct vulnerability indicators and precise mechanisms is still needed. If we understand which characteristics are indicative of vulnerability, preventive approaches may be enhanced.

Both the traditional vulnerability-stress-models (e.g., [2]) and their more recent cognitive extensions [4] ascribe to the state of hyperarousal a central role in psychotic symptom formation. According to the cognitive model of persecutory delusions, vulnerability will cause individuals to respond to stressful events by a stronger increase in arousal than can be expected for nonvulnerable individuals. This arousal may be further intensified and prolonged by additive factors such as sleep-disturbances or long-term anxiety [4]. This hyperarousal of vulnerable individuals may then cause an inner-outer confusion leading to anomalous perceptual experiences such as hearing thoughts as voices. Individuals will consequently search for a meaning of their arousal and of these experiences, nurturing the threat belief [4]. In support of the role of high arousal in symptom formation, different measures of trait reactivity have been found to interact with life events to predict psychotic symptoms [5]. Also, an increased sensitivity to stressors was found not only to precede symptom exacerbations and relapse in patients with psychosis [6] but also to be characteristic of individuals at ultrahigh risk for psychosis [7, 8]. Over and above sensitivity to minor stressors, a generally elevated level of arousal is also characteristic of individuals with psychosis [9]. Thus, hyperarousal could be a promising indicator of vulnerability to psychosis.

Researchers have proposed a personality trait called arousal predisposition (AP) that combines aspects of trait and stimuli processing and can readily be assessed by self-report scales [10, 11]. It is defined as the extent to which a person responds to heightened complexity, variation, and/or unexpectedness of stimuli with increased arousal [11]. Hence, it describes a person's responsiveness to the environment [10]. In line with the above definition, AP was found to be associated with larger electromyographic and electrodermal activity after the exposure to novel stimuli (e.g., white noise), thus with increased autonomic arousal responses [10]. In contrast to neuroticism, which is a more complex trait that involves characteristic unpleasant responses to situations and specific negative emotional and cognitive experiences such as guilt, AP is narrowly defined and proposed to be a possible foundation for later neuroticism (see [11, 12]). It describes the perception of immediate sensory (e.g., “I find that my heart keeps beating fast”) and emotional (e.g., “I can be emotionally moved”) responses rather than involving complex levels of secondary appraisals [11, 12]. Two studies found AP to be increased in participants with schizophrenia compared to healthy controls [5, 13]. The same group of researchers also reported a positive association of AP and positive and affective symptom severity [13]. Furthermore, they added support to the construct validity by demonstrating that, in the participants with schizophrenia, AP was significantly associated with the self-reported stress related to a speech stress condition [13]. The authors concluded that heightened AP increases an individual's stress-responsiveness and speculated that this could be a cause of stress-related symptom exacerbation. However, as the authors point out themselves, the increased level of AP and thus stress-responsiveness may also be a result rather than a precursor of the mental disorder [13]. Furthermore, results from later studies have been inconclusive [12] and the authors emphasized the need to examine whether and under which circumstances the association of psychotic symptoms and stress-responsiveness assigned to AP may be found. Taken together, previous findings assign AP a role in explaining psychotic symptoms but replication and further exploration are needed to corroborate the proposed assumptions and to clarify whether AP is indeed a valid indicator of vulnerability to psychosis.

Following the assumption that AP is a risk factor for the development of psychotic symptoms and that both psychotic symptoms and their risk factors occur on a continuum [14], an association between AP and symptoms should already be present before symptoms reach clinical relevance. Consequently, one would expect to find the association in any kind of population sample.

**Figure 1:**
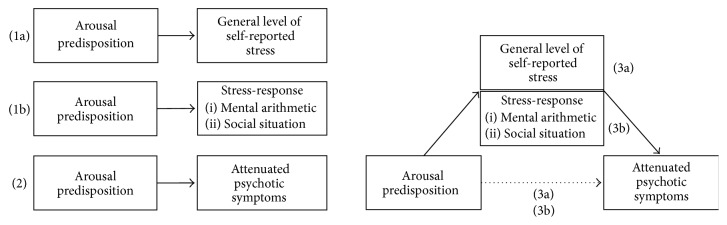
Graphical depiction of the hypotheses.

Thus, we expect individuals without a psychotic disorder but with high AP to (1a) report higher general levels of stress and (1b) show an increased response to discrete stressors (i.e., stress-response) compared to individuals with low AP. Furthermore, if AP constitutes a vulnerability factor for psychotic symptoms, we expect that (2) AP will be positively associated with the extent of subclinical psychotic symptoms. Finally, we hypothesize that the association of AP with symptoms is mediated by general levels of self-reported stress (3a) and the stress-responses (3b; see Figure 1).

## 2. Materials and Methods

### 2.1. Design and Procedure

**Figure 2:**
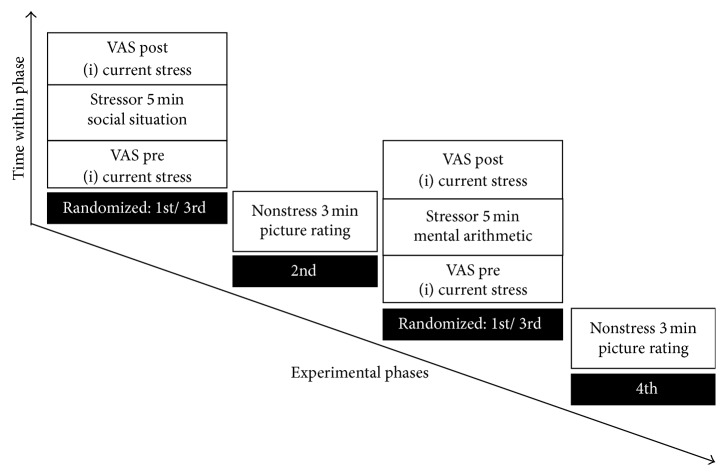
Procedure of the stress induction. VAS: Visual Analogue Scales. “1st/3rd”: randomized order of the phases.

We conducted the study within the general population as an online study and included a stress induction to measure the stress-response. Before the questionnaires and tasks, participants were told to switch off any possible source of distraction (e.g., music), to close all other browsers, and to concentrate on the participation. Then, sociodemographic data, psychotic symptoms, and AP were assessed, followed by the stress induction (see Figure 2).

Psychotic symptoms were measured with the German version of the Community Assessment of Psychic Experiences (CAPE) questionnaire [15]. Participants rated their experiences during the previous four weeks on a 4-point scale from “never” to “nearly always.” The self-report scale assesses positive symptoms with 20 items, negative symptoms with 14 items, and symptoms of depression with eight items. A total score for overall psychotic symptoms is calculated from all three subscales. Good reliability and validity have been reported [16].

AP was measured with the Arousal Predisposition Scale (APS) [17]. The 12-item self-report scale incorporates a self-descriptive format, such as “I can be emotionally moved by what other people consider to be simple things,” “I get excited easily,” and “I am restless and fidgety.” For the present study, the APS was translated into German. A back translation was analyzed for concordance with the original version. All discrepancies of the new English version from the original one were due to the use of synonyms. Participants rated their consent with the statements from “1 = never” to “5 = always” and a total score was calculated. The internal consistency of the English version has been found to be acceptable or good [17, 18]. Stability, as intended by a trait index, was found by a retest correlation of .65 in a 9-month follow-up [5].

For the stress induction, participants were exposed to two different 5-minute stressors (an arithmetic stressor and a social stressor) in randomized order. This procedure was adapted from previous studies investigating stress-responses in patients with schizophrenia [13, 19]. The arithmetic stressor required mental arithmetics that were adapted from an intelligence test battery to increase the stressor's potential (e.g., 60 − 25 = *A*. *A* = ?; 2/3*∗*75 + 1/3*∗*60 = *F*. *F* = ?). Twenty-five arithmetic problems were displayed at the same time for the duration of five minutes, allowing participants to skip forward and backward. Engagement in the task was tested by reviewing the typed responses. The social stressor required the description of an every-day stressful social interaction. (“Please recall the situation and describe it in as much detail as possible. The situation should have been stressful and occurred within an every-day social context.”). The participants typed in the situation within the 5-minute period, after which the next screen occurred. Similar to the arithmetic stressor, engagement in the task was considered by reviewing the typed responses. Each stress condition was followed by a 3-minute nonstress condition (18 pictures; e.g., objects and landscapes). During the nonstress conditions participants were kept committed to the experiment by rating the extent to which the pictures elicited emotions in them via Visual Analogue Scales (VAS) based on Gaab et al. [20], ranging from “not at all” to “very intensely.” Before and after the stress tasks, VAS were employed to assess subjective stress with the item “I feel stressed by the situation.” The stress-response was defined as the increase in stress from pre- to poststressor assessment. Furthermore, another VAS at the end of the experiment assessed the participants' subjective impression of their ability to concentrate throughout the experiment. All VAS scrollable bars had 100 invisible increments.

### 2.2. Participants

Participants were recruited through flyers at different universities, social networks, and Internet platforms (e.g., psychology or stress forums). Students from the authors' university were granted partial course credit after obtaining the link via an electronic credit system.

Participation was voluntary and could be stopped at any point without explanation. Anonymity of data was guaranteed as no codes, names, or residencies were assessed. In the beginning and at the end of the study, participants were given a contact address for any requests or problems that occurred before, during, or after the study. After being welcomed and thoroughly informed, participants had to explicitly give consent by clicking “continue with participation.”

Previous research in clinical samples showed a medium sized correlation of AP with positive symptoms (*r* = .321) [13]. Thus, we based the power calculation on the expectation of medium effect sizes (*ρ* = .30 and *f* = .25). With *α* = .05 and a power of .90, the calculations with G*∗*Power [21] yielded a required sample size of *N* = 46 for repeated-measures within-between interaction (two groups, two measurements, correlation among measurements *r* = 0.5), of *N* = 92 for one-tailed bivariate correlations, and of *N* = 88 for a linear multiple regression analysis.

In total, 184 participants agreed to participate in the study. No adverse events were reported to the provided contact. After excluding the participants for whom a lack of engagement was evident (i.e., who did not complete the questionnaires, *n* = 57, or participate in both stressors, *n* = 23), 104 participants constituted the final sample. The reported mean age was 27.7 years (*SD* = 11.2, range 18–70). Of these participants, the majority was female (*n* = 74, 71.2%) and well educated (*n* = 73, 70.2% with 10–13 years of education and *n* = 31, 29.8% with a university degree). Furthermore, 15 participants (14.4%) reported a diagnosed mental disorder (the majority of these being depression and anxiety disorders) and 8 participants (7.7%) were in treatment at the time of participation. No participant reported having a psychotic disorder.

### 2.3. Data Analysis

To analyze the impact of the extent of AP for the stress-levels (hypothesis 1a) and stress-response (hypothesis 1b), we divided the sample into terciles according to the APS scores. For a better distinction from two extreme ends, the upper and lower terciles were regarded as between-subject variable and analyzed in repeated-measures ANOVA for pre- and poststress, distinctively for the two stressors. Bivariate correlations were conducted to verify the proposed association between the CAPE and the APS (hypothesis 2). After confirming the hypotheses (1a) or (1b) and (2), we planned to test the association between stress-level, stress-response (i.e., difference value post-pre), and symptoms using bivariate correlations and the mediation hypotheses (3a and 3b) using linear multiple regression models.

Prior to the analyses, the scale distributions were tested for normality with the Kolmogorov-Smirnov test and visually checked for outliers within a stem-and-leaf plot. Due to directed hypotheses, the correlations were conducted one-tailed. The level of significance was determined as *p* ≤ .05. All analyses were conducted with SPSS version 20.

## 3. Results

### 3.1. Data Quality and Manipulation Check

Both the core scales of CAPE total and APS total met the requirements of normal distribution (*p* ≥ .61). In the stem-and-leaf plots, three outliers (*n* = 2 for CAPE and *n* = 1 for APS) were identified and excluded in subgroup analyses (see Section 3.5). The stress ratings were not normally distributed and showed several outliers. However, in the total and sufficiently large sample, the standard parametric testing was assumed to be robust.

The manipulation of the stress-levels was successful. For the arithmetic stressor, the mean poststress-level (VAS: *M* = 46.5 and *SD* = 28.1) was significantly higher than the prestress-level (VAS: *M* = 32.2 and *SD* = 28.8), *t*(103) = −5.215, *p* < .001, and Cohen's *d* = 0.5. The participants' responses revealed a mean of 12.5 (*SD* = 4.5) correct answers, with a range from 2 to 25. Only two participants gave less than five correct answers (i.e., one per minute) and exclusion of these participants did not change the reported results. Similarly, for the social stressor, the poststress VAS scores (*M* = 37.8 and *SD* = 29.5) were significantly higher than the prestress VAS scores (*M* = 31.2 and *SD* = 26.7), *t*(103) = −4.026, *p* < .001, and Cohen's *d* = 0.40. A review of the content of the stated situations by the first author revealed that two participants did not describe a distinct social situation and four participants did not provide sufficient detail (i.e., only one or two short sentences). However, the exclusion of these six participants did not change the results reported in the main findings. Furthermore, the prestress VAS levels did not differ between the two stress conditions, indicating that the nonstress conditions in between were successful in reestablishing baseline values, *t*(103) = −0.505 and *p* = .614. In line with that, the pictures that we used for the nonstress condition were rated low in the VAS for eliciting emotions; the ratings after the social stressor, *M* = 29.0 and *SD* = 17.2, and after the arithmetic stressor, *M* = 28.7 and *SD* = 17.0, did not differ significantly, *t*(102) = −0.365 and *p* = .716.

Finally, the participants' overall concentration on the tasks was rated with a mean of 68.8 (*SD* = 25.4), indicating acceptable concentration. Including concentration as a covariate did not change the reported results.

### 3.2. Construct Validity: Hypotheses (1a) and (1b)

The two APS groups that were used for the analyses consisted of *n* = 31 participants in the lower tercile (i.e., APS_low_) and *n* = 35 participants in the upper tercile (i.e., APS_high_). Within the possible range of 12–60, APS_low_ showed a mean total score of 24.1 (SD = 2.9, range 18–28) and APS_high_ of 40.4 (SD = 4.7, range 35–56).

**Figure 3:**
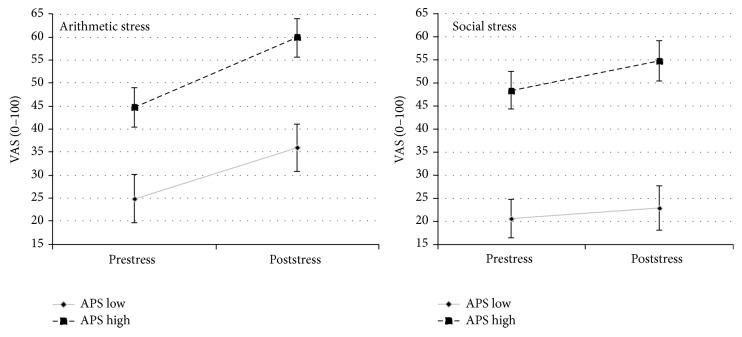
Repeated-measures ANOVA for stress-response in low versus high APS groups. APS: Arousal Predisposition Scale; VAS: visual analogue stress scales.

The repeated-measures ANOVAs for the arithmetic stressor showed a significant main effect of group, *F*(1, 64) = 14.67, *p* < .001, and *η* ^2^_*p*_ = .186, with APS_low_ revealing lower general stress estimates than APS_high_. Similarly, for the social stressor, a main effect of group was found, *F*(1,64) = 25.33, *p* < .001, and *η* ^2^_*p*_ = .284, confirming hypothesis (1a) (see Figure 3).

Contrary to hypothesis (1b), no significant time × group interaction was found, neither for the arithmetic, *F*(1, 64) = 0.34, *p* = .564, and *η* ^2^_*p*_ = .005, nor for the social stressor, *F*(1, 64) = 1.23, *p* = .272, and *η* ^2^_*p*_ = .019 (see Figure 3).

### 3.3. Arousal Predisposition as a Vulnerability Factor: Hypothesis (2)

As reported in Table 1, there was a significant correlation between the CAPE symptoms and the APS score. Also, all three CAPE subscales were significantly associated with the APS.

### 3.4. Stress as a Potential Mediator: Hypotheses (3a) and (3b)

There was a significant association of the CAPE symptoms with the sum of the prestressor stress-levels (*r* = .35 and *p* < .001). The regression model for hypothesis (3a) revealed a decrease in the *β*-weight when the stress-level was also considered. However, the change in *R* ^2^ and the *β*-weight for the model including stress-level showed a nonsignificant trend only, while the APS was still a significant predictor for the mean score in the CAPE (see Table 2).

The present data did not confirm hypothesis (1b). Thus, the mediation hypothesis (3b) was not pursued further.

### 3.5. Exploratory Analyses of Potential Confounders

A potential confounding of age was investigated by including age as a covariate in partial correlations of APS with stress-levels and CAPE as well as in the regression analysis of APS and stress-level predicting symptoms. However, this did not affect any of the results.

To investigate the generalizability of the effects further, we conducted several subgroup analyses (i.e., subsample without the outliers, male participants and female participants only, and participants without reported mental disorders). The following slightly different patterns of results emerged: (a) for the male participants hypothesis (1a) was not confirmed for the arithmetic stressor (*n* = 17 and *p* = .328) and hypothesis (2) was not confirmed for the CAPE subscales for negative and depressive symptoms, and (b) for the subsamples without mental disorders and without outliers, the regression models to test hypothesis (3) were significant for APS as a predictor for subclinical symptoms and also showed a significant predictive value for the general stress-level (*p* ≤ .040). All other results remained unchanged.

## 4. Discussion

The present study investigated whether the construct of a predisposition to arousal is an indicator of vulnerability to psychosis. We expected that individuals with high arousal predisposition would report higher general stress-levels, show an increased response to stressors, and have higher levels of psychotic symptoms compared to individuals with low arousal predisposition. To clarify possible mechanisms, we analyzed whether the latter association is mediated by the general stress-level or the stress-response.

As expected we found that the group with increased arousal predisposition scores reported higher general levels of subjective stress, before as well as after the stressor. This is in line with studies showing increased autonomic arousal and increased subjective stress-estimates, even at rest, for individuals with clinical psychosis [22]. Previously, this was not found for a group with attenuated psychotic symptoms [22]. Importantly, these findings are not a result of the extreme group approach we used in the study as the correlations between general, pre-, and poststress-levels with arousal predisposition were also significant (all *r* > .32, *p* < .001). Also, in exploratory analyses, the results were robust to differences in the engagement in the task or the presence of a mental disorder. However, some associations were no longer significant in the group of the male participants only. Even though this may be attributed to a lack of power for this analysis, further investigations of gender differences in stress-responses are warranted. Concluding, the Arousal Predisposition Scale seems to be a promising indicator of vulnerability beyond clinical symptoms.

Contrary to what we expected from a trait that describes a person's responsiveness to the environment [10], we did not find arousal predisposition to be associated with the distinct responses to either of the stressors. Thus, arousal predisposition was only relevant to the subjective perception of “generally feeling stressed.” At first glance, this appears surprising because previous studies found an association of arousal predisposition with stress-levels [13] and negative affect [12] after the exposure to stress. However, a closer look reveals that in the studies by Dinzeo et al. [12, 13] this was evident only for the participants with schizophrenia, not for their healthy controls. More importantly, in one study [13] the prestress values were not considered, making it difficult to ascertain whether the association refers to the general level of self-reported stress or to the distinct stress-response. Also in contrast to the expected validity of the construct, Docherty et al. [5] did not find arousal predisposition to interact significantly with life events to predict symptoms. They did, however, show an interaction of other reactivity measures such as emotional reactivity and trait anxiety with stressors to increase symptoms. Following from these findings, they propose that the items of the arousability predisposition scale emphasize the reactivity to sensory stimuli rather than to social stimuli [5]. This could be another explanation for the nonsignificant association of the predisposition to arousal with the online stressors in our study. Furthermore, there may have been ceiling effects due to the generally elevated stress-levels. It is also important to note that responses to our stressors were very heterogeneous. Our design may have left a wide range of ways to engage in the task. One could speculate that participants who are easily aroused may have been more tempted to avoid high engagement with the stressor than participants with low arousability. In sum, arousal predisposition seems to be related to the general level of subjective stress. The predictive value for the stress-response may be limited to distinct stressors only, may occur in more controlled or natural situations only, and may be stronger in clinical samples.

As hypothesized, we found the degree of arousal predisposition to be positively and moderately associated with the extent of psychotic symptoms. This finding is consistent with research in clinical psychosis that showed an association of arousal predisposition with positive and affective symptom severity of a similar extent [13]. It also confirms the importance of high arousal for symptom formation that has been proposed before [2, 23]. The fact that we found this association to be present in a population sample with only subclinical psychotic symptoms adds further support to the assumption that arousal predisposition may be a vulnerability indicator for psychosis.

In our mediation analyses, we were able to replicate the known association between general levels of stress and psychotic symptoms (e.g., [8, 24]). However, neither general levels of stress nor stress-sensitivity explained the association between arousal predisposition and symptoms. Excluding outliers and participants with mental disorders even corroborated this finding, with both arousal predisposition and general stress-levels predicting the occurrence of symptoms but not explaining the association. Future research should attempt to further elucidate the mechanisms that could explain why arousal predisposition may be predictive of psychotic symptoms. A possible mechanism could be that characteristic cognitive biases may lead to a premature interpretation of the increased arousability. In turn, this may enhance the formation of a threat belief as stated in cognitive models of persecution [4]. Moreover, by leading to an increase in emotional and physical responsiveness, arousal predisposition may interact with a decreased ability of emotion regulation and less efficient recovery after stress. In order to investigate the validity of this hypothesized causal chain, longitudinal observation of arousal predisposition, emotion regulation, and stress-sensitivity (emotionally and physically) is required, most efficiently within natural environments.

To our knowledge, the present study is the first to investigate arousal predisposition as a trait related to vulnerability for psychosis. Arousal predisposition is easily accessible via self-report, which has clear advantages compared to other possible predictors such as psychophysiological measurements. However, the study has some shortcomings that should be amended in future research. Firstly, the proposed association to subclinical symptoms needs to be confirmed in a more representative sample of the general population, as our sample mainly consisted of female, highly educated, and young participants, limiting its generalizability. Secondly, the predictive value of arousal predisposition for the stress-response should be approached in real-life assessments of stress. In the online stress induction we were not able to prevent external disturbances or avoidance behavior which may have influenced the task, leading to a variance of possibilities to engage in it. In addition, for future online studies, it might be helpful to further ensure continuous engagement by adapting the tasks (e.g., timing each arithmetic problem separately). Thirdly, one could argue that an acquiescence bias may have led to the positive associations, as few items were reversely coded. However, none of the participants' scores yielded a particular or systematic answering pattern. Future research should also include a follow-up on symptom change to disentangle causality. If it can be shown that arousal predisposition particularly predicts psychotic symptoms and response to stress in a longitudinal design, this would support its value as a vulnerability indicator. Moreover, the specificity of the association of increased arousal predisposition with psychosis in comparison to other disorders is interesting, because a strong association to depressive symptoms was shown in our study and in previous research [12].

## 5. Conclusions

In the present study, we found that individuals with higher levels of subclinical psychotic symptoms also showed a higher predisposition to arousal. Thus, the predisposition may be a relevant indicator of vulnerability to psychosis. We also found that arousal predisposition was associated with general levels of self-reported stress. However, it was not associated with specific responses to discrete stressors. A clinical implication of the presented findings is the need to detect and target elevated stress-levels early. In line with this, the increase of stress-resilience has been proposed as a possible goal in psychosis therapy (e.g., [6, 25]) and early at-risk interventions (e.g., [26]). Taken together with our finding that an increased predisposition to arousal is already indicative of subclinical psychotic symptoms, early intervention modules within stress-management programs seem promising and could potentially target hyperarousal specifically (see also [9]). Further studies are needed to confirm the value of arousal predisposition as an indicator of vulnerability to psychosis and its potential to enhance the development and implementation of stress-resilience trainings.

## Figures and Tables

**Table 1:** Pearson's correlation coefficients, psychometric data, and scale properties.

Measure	1	2	3	4	*M (SD) *	*α*
(1) APS total score	—				32.4 (7.3)	.83

(2) CAPE total score	.51^*∗∗∗*^	—			69.8 (10.4)	.87
(3) CAPE positive	.35^*∗∗∗*^	.66^*∗∗∗*^	—		27.6 (4.1)	.71
(4) CAPE negative	.28^*∗∗*^	.87^*∗∗∗*^	.34^*∗∗∗*^	—	26.8 (5.9)	.86
(5) CAPE depressive	.60^*∗∗∗*^	.71^*∗∗∗*^	.23^*∗*^	.47^*∗∗∗*^	15.5 (3.7)	.84

*Note. N* = 104; *α* = Cronbach's *α*; CAPE: Community Assessment of Psychic Experiences; APS: Arousal Predisposition Scale.

^*∗*^ *p* < .05. ^*∗∗*^ *p* < .01. ^*∗∗∗*^ *p* < .001, one-tailed.

**Table 2:** Multiple regression analyses predicting psychotic symptoms from arousal predisposition and general levels of stress.

Predictor	CAPE total score		
	Δ*R* ^2^	*β*	*p*
Step 1	.256		<.001
APS total score		.506	<.001

Step 2	.021		.093
APS total score		.438	<.001
VAS stress-level		.159	.093

Total *R* ^2^	.277		<.001

*Note. N* = 104; CAPE: Community Assessment of Psychic Experiences; APS: Arousal Predisposition Scale; VAS: Visual Analogue Scales (here: mean of the prelevels before both stressors).
